# Prevalence and prognosis of respiratory pendelluft phenomenon in mechanically ventilated ICU patients with acute respiratory failure: a retrospective cohort study

**DOI:** 10.1186/s13613-022-00995-w

**Published:** 2022-03-05

**Authors:** Yi Chi, Zhanqi Zhao, Inéz Frerichs, Yun Long, Huaiwu He

**Affiliations:** 1 State Key Laboratory of Complex Severe and Rare Disease, Department of Critical Care Medicine, Peking Union Medical College Hospital, Peking Union Medical College, Chinese Academy of Medical Sciences, 1 shuaifuyuan, Dongcheng District, Beijing, China; 2 Department of Biomedical Engineering, Fourth Military Medical University, Xi’an, China; 3 Institute of Technical Medicine, Furtwangen University, VS-Schwenningen, Germany; 4 Department of Anesthesiology and Intensive Care Medicine, University Medical Center of Schleswig-Holstein Campus Kiel, Kiel, Germany

**Keywords:** Pendelluft, Mechanical ventilation, Acute respiratory failure, Intensive care unit

## Abstract

**Background:**

Respiratory pendelluft phenomenon, defined as intrapulmonary gas redistribution caused by asynchronous alveolar ventilation, could be potentially harmful by inducing lung injury. The aim of the present study was to investigate its prevalence and prognosis in intensive care unit (ICU) patients with acute respiratory failure (ARF).

**Methods:**

This was a retrospective observational study on 200 mechanically ventilated ARF patients treated in a tertiary ICU. The presence of pendelluft was determined using electrical impedance tomography (EIT) within 48 h after admission. Its amplitude was defined as the impedance difference between the sum of all regional tidal impedance variation and the global tidal impedance variation. A value above 2.5% (the 95th percentile from 30 healthy volunteers) was considered confirmative for its occurrence.

**Results:**

Pendelluft was found in 61 patients (39 in 94 patients with spontaneous breathing, 22 in 106 receiving controlled ventilation), with an overall prevalence of 31%. Existence of spontaneous breathing and higher global inhomogeneity index were associated with pendelluft. Patients with pendelluft had a longer ICU length of stay [10 (6, 14) vs. 7 (4, 11) days; median (lower, upper quartile); *p* = 0.022] and shorter 14-day ventilator-free days [8 (1, 10) vs. 10 (6, 12) days; *p* = 0.015]. Subgroup survival analysis suggested the association between pendelluft and longer ventilation duration, which was significant only in patients with PaO_2_/FiO_2_ ratio below 200 mmHg (log-rank *p* = 0.042). ICU mortality did not differ between the patients with and without pendelluft.

**Conclusions:**

Respiratory pendelluft occurred often in our study group and it was associated with longer ventilation duration. Early recognition of this phenomenon should trigger interventions aimed at alleviating pendelluft.

**Supplementary Information:**

The online version contains supplementary material available at 10.1186/s13613-022-00995-w.

## Introduction

Respiratory pendelluft is the phenomenon of intrapulmonary gas redistribution caused by asynchronous alveolar ventilation. It has been spotted in patients with flail chest [1], obstructive lung disease [2], and acute respiratory distress syndrome (ARDS) [3]. Evidence from animal experiments has suggested that pendelluft could be potentially harmful by inducing local overdistension and tidal recruitment [4–6]. Hence, the early recognition of pendelluft is warranted for timely adjustment of treatment and ventilation strategy, especially in critically ill patients. Earlier techniques (positron imaging, multi-channel lung sound analysis, darkfield microscopy, etc.) used to monitor pendelluft are not suitable for critically ill patients.

The chest electrical impedance tomography (EIT) is a non-invasive monitoring technique that can obtain real-time images of regional lung ventilation at the bedside by detecting the bio-impedance changes during consecutive respiratory cycles [7]. The application of EIT has enabled bedside detection of respiratory pendelluft in patients in the intensive care unit (ICU). It allows not only qualitative, but also quantitative analysis of pendelluft [8, 9]. Despite the progress, little is known about the epidemiology of pendelluft and its association with clinical outcome.

The primary objective of this retrospective study was to explore the prevalence and prognosis of pendelluft in mechanically ventilated ICU patients with acute respiratory failure (ARF).

## Methods

From January 2020 to November 2021, ICU patients with ARF, defined by the ratio of partial pressure of arterial oxygen to fraction of inspired oxygen (PaO_2_/FiO_2_) below 300 mmHg within 48 h of admission, and receiving mechanical ventilation were eligible for study inclusion. The exclusion criteria were: age < 18 years, pregnancy, body mass index over 50 kg/m^2^, ribcage malformation, and any contraindication to the use of EIT (automatic implantable cardioverter defibrillator, chest wounds limiting electrode belt placement, implantable pumps, etc.). This retrospective study was approved by the Institutional Research and Ethics Committee of Peking Union Medical College Hospital (S-K1859).

The following parameters were documented: age, sex, predicted body weight (PBW), Acute Physiology and Chronic Health Evaluation II (APACHE II) score, arterial blood gas and respiratory parameters at the time point of EIT recording [tidal volume, respiratory rate, positive end-expiratory pressure (PEEP), existence of spontaneous breathing (defined as EIT-based respiratory rate higher than set respiratory rate under controlled ventilation)], ventilatory ratio (minute ventilation [mL/min] × PaCO_2_ [mmHg])/(PBW [kg] × 100 [mL/min] × 37.5 [mmHg]) [10], outcome measures such as ICU length of stay, 14-day ventilator-free days (VFD) and ICU mortality.

### EIT-based measurements

EIT measurements were performed within 24 h of proven ARF diagnosis with PulmoVista 500 (Dräger Medical, Lübeck, Germany). An EIT belt with 16 electrodes was placed around the patient’s thorax at the 4–5th intercostal space level. EIT measures changes in voltages between electrode pairs, reconstructs the impedance changes within the measurement plane and then calculates the regional ventilation map by subtracting the inspiration-begin from the expiration-begin image as well as the global tidal signal variation.

To establish a reference value, EIT measurements were performed in 30 healthy volunteers without any underlying lung disease (demographics are summarized in Additional file 1: Table S1). The following EIT-based parameters were calculated in both patients and healthy subjects:

#### Pendelluft

A recent published EIT-based algorithm for pendelluft detection [9] was adopted by our study. According to the theory of pendelluft proposed by Otis et al. [11], when pendelluft occurs, the sum of the regional tidal volumes is greater than the overall tidal volume, their difference representing the pendelluft volume. Similarly, the EIT-based pendelluft amplitude was defined as the impedance difference between the sum of all regional tidal impedance variation (TIV) and the global TIV (Additional file 1: Fig. S1). Since this pixel-based algorithm is so sensitive, the occurrence of pendelluft was considered only when its amplitude exceeded 2.5% of global TIV (which was the 95th percentile from 30 healthy volunteers) (Additional file 1: Table S1).

#### Ventilation defect score

Its calculation was based on a semi-quantitative method for analyzing heterogeneity of ventilation distribution validated by a previous study [12]. The global ventilation map was separated into four non-overlapping quadrants of equal size to trace gas distribution into different regions of interest (ROIs): lower left (LL), lower right (LR), upper left (UL) and upper right (UR). Distribution defects in each quadrant were scored as follows: 0 (quadrant distribution% ≥ 15%), 1 (15% > quadrant distribution% ≥ 10%) and 2 (quadrant distribution% < 10%). The total ventilation defect score was the sum of the scores of each quadrant (Additional file 1: Fig. S2).

#### Global inhomogeneity (GI) index

The GI index was designed to describe the overall degree of spatial heterogeneity of ventilation [13]. A smaller global inhomogeneity index represents a more homogeneous distribution.

### Statistical analysis

Descriptive data are expressed as numbers and percentages for categorical variables and median (lower, upper quartile) for continuous variables. Categorical variables were compared using the Pearson Chi-square test, whereas continuous variables distributed nonparametrically between groups were compared using the Mann–Whitney *U* test. Pendelluft amplitudes among groups of different defect scores were compared using the Kruskal–Wallis *H* test.

To evaluate independent factors associated with pendelluft, significant univariate risk factors or variables considered clinically relevant to pendelluft were examined using backward stepwise multivariable logistic regression analysis. To avoid overfitting, a maximal number of six variables in the pendelluft model was entered in view of the 61 events observed (APACHE II score, PaO_2_/FiO_2_ ratio, PEEP, spontaneous breathing, respiratory rate and GI index). The linear relationship of PEEP and pendelluft amplitude was explored with Spearman correlation coefficient. To analyze the relationship between pendelluft and ventilation duration, cause-specific Cox proportional hazard models were implemented to predict the relative hazard of successful discontinuation from ventilator with 95% confidence intervals; the model was adjusted for APACHE II score and PaO_2_/FiO_2_ ratio. Restricted cubic spline was used to explore the possible nonlinear relationship between pendelluft amplitude and the relative hazard of discontinuation from ventilator. P values of less than 0.05 were considered statistically significant. Statistical analyses were performed using SPSS 25.0 (SPSS, Chicago, IL) and R version 4.0.3.

## Results

### Risk factors for pendelluft

**Fig. 1:**
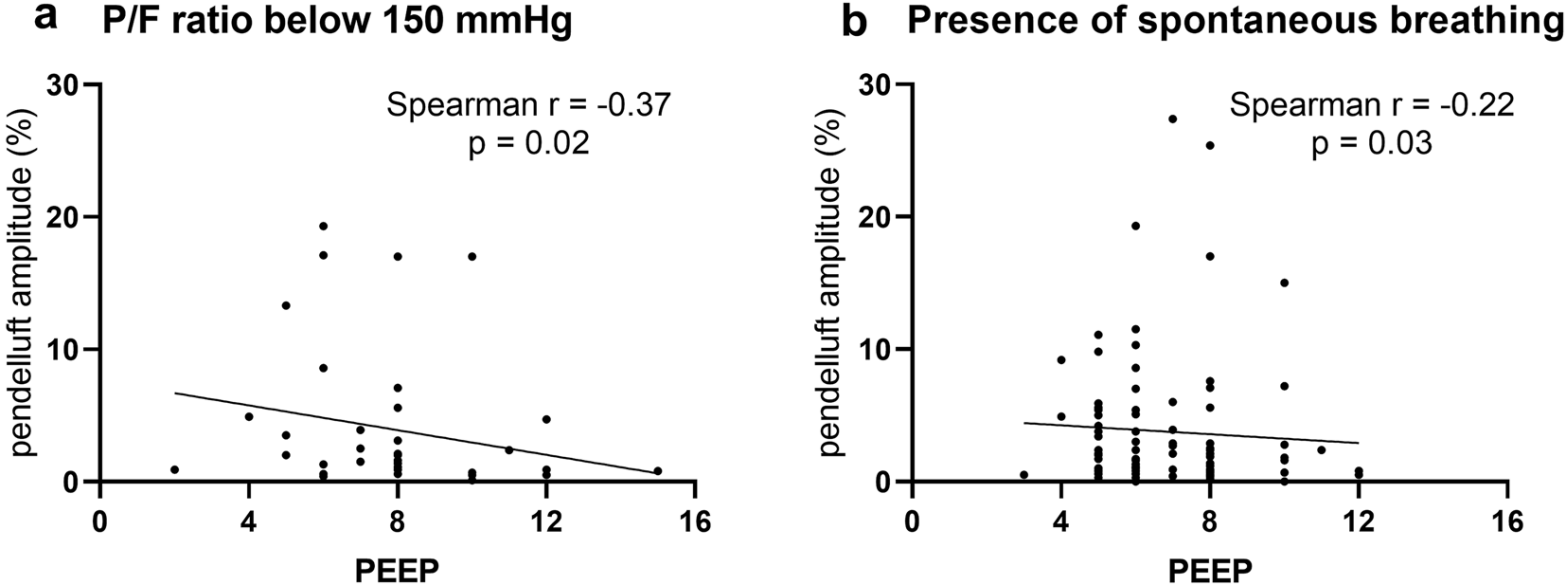
Exploration of correlation between PEEP and pendelluft amplitude in **A** P/F ratio below 150 mmHg and **B** presence of spontaneous breathing, respectively

A total of 200 patients (135 men and 65 women) were included, with a median age of 62 years and a median APACHE II score of 16 at 24 h of ICU admission. Patients received mechanical ventilation with a median PaO_2_/FiO_2_ ratio of 200 (164, 246) mmHg, tidal volume of 428 (396, 501) mL, PEEP of 7 (5, 8) cmH_2_O, and respiratory rate of 17 (15, 19) cycles/minute at the time point of EIT recording (Table 1). 94 (47%) of them had spontaneous breathing. Pendelluft was detected in 61 of the 200 ARF patients (prevalence of 31%), 39 of 94 spontaneously breathing patients (41%) and 22 of 106 patients receiving fully controlled ventilation (21%). Patients with pendelluft had a higher proportion of spontaneous breathing, respiratory rate and higher GI index (Table 1). Tidal volume, PEEP, PaCO_2_ and ventilatory ratio did not differ between the groups. Multivariable logistic regression analysis identified that the existence of spontaneous breathing and higher GI index were associated with pendelluft (Table 2). The relationship between PEEP and pendelluft amplitude was explored in one subgroup of patients with P/F ratio below 150 mmHg (39 patients; Spearman r = − 0.37, *p* = 0.02) and the other subgroup of spontaneous breathing patients (94 patients; Spearman r = − 0.22, *p* = 0.03) (Fig. 1).

**Table 1:** Clinical characteristics and outcomes of patients having acute respiratory failure with or without pendelluft

	All patients (*n* = 200)	No pendelluft (*n* = 139)	Pendelluft (*n* = 61)	*p* value
Age, years	62 (51, 69)	62 (51, 68)	62 (50, 73)	0.549
Female gender, *n* (%)	65 (32%)	44 (32%)	21 (34%)	0.825
Predicted body weight, kg	64.0 (56.2, 68.5)	64.0 (54.1, 68.5)	64.9 (56.8, 69.4)	0.290
APACHE II score	16 (13, 20)	16 (13, 20)	17 (14, 23)	0.153
Respiratory parameters				
Spontaneous breathing, *n* (%)	94 (47%)	55 (40%)	39 (64%)	0.002
Tidal volume, mL	428 (396, 501)	430 (398, 500)	421 (390, 510)	0.810
Respiratory rate, minute^−1^	17 (15, 19)	16 (15, 18)	18 (16, 21)	0.002
PEEP, cmH_2_O	7 (5, 8)	7 (5, 8)	6 (5, 8)	0.152
Arterial blood gas				
PaO_2_/FiO_2_ ratio, mmHg	200 (164, 246)	204 (167, 249)	194 (151, 239)	0.225
pH	7.4 (7.4, 7.5)	7.4 (7.4, 7.5)	7.4 (7.4, 7.5)	0.763
PaCO_2_, mmHg	40.8 (37.0, 43.9)	41.0 (37.6, 44.0)	39.4 (36.0, 42.4)	0.189
Ventilatory ratio	1.3 (1.2, 1.5)	1.3 (1.2, 1.5)	1.4 (1.2, 1.6)	0.170
EIT parameters				
Dorsal ventilation, %	41 (32, 47)	41 (32, 48)	41 (32, 47)	0.984
Defect Score, *n* (%)				0.042
0	88 (44%)	68 (49%)	20 (33%)	
1	61 (30%)	42 (30%)	19 (31%)	
≥ 2	51 (26%)	29 (21%)	22 (36%)	
GI index	0.36 (0.34, 0.38)	0.36 (0.34, 0.37)	0.37 (0.34, 0.39)	0.023
Pendelluft amplitude, %	1.4 (0.7, 3.5)	0.9 (0.5, 1.6)	6.4 (3.9, 13.3)	< 0.001
Outcome				
MV duration, days	5 (3, 8)	4 (2, 7)	6 (3, 8)	0.052
ICU length of stay, days	7 (5, 13)	7 (4, 11)	10 (6, 14)	0.022
14-day ventilator-free days	9 (5, 11)	10 (6, 12)	8 (1, 10)	0.015
ICU mortality, *n* (%)	23 (12%)	13 (9%)	10 (16%)	0.151

Data are presented as median (lower, upper quartile) unless otherwise specified

*APACHE* Acute Physiology and Chronic Health Evaluation, *PEEP* positive end-expiratory pressure, *EIT* electrical impedance tomography, *GI* global inhomogeneity, *MV* mechanical ventilation, *ICU* intensive care unit

**Table 2:** Univariate and multivariate logistic regression analysis for pendelluft

Variables	Univariate logistic regression		Multivariate logistic regression	
	Odds ratio (95% CI)	*p*	Odds ratio (95% CI)	*p*
APACHE II score	1.043 (0.994–1.096)	0.086	1.053 (0.999–1.111)	0.052
PaO_2_/FiO_2_ ratio	0.997 (0.991–1.002)	0.211	0.995 (0.989–1.002)	0.164
PEEP	0.930 (0.792–1.082)	0.357	0.944 (0.789–1.123)	0.520
Spontaneous breathing	2.707 (1.463–5.112)	0.002	2.375 (1.175–4.883)	0.017
Respiratory rate	1.133 (1.044–1.235)	0.003	1.070 (0.975–1.178)	0.157
GI index (per 0.01 increase)	1.153 (1.049–1.275)	0.004	1.141 (1.030–1.272)	0.014

*APACHE* Acute Physiology and Chronic Health Evaluation, *PEEP* positive end-expiratory pressure; *GI* global inhomogeneity

Higher proportion of higher ventilation defect score was seen in patients with pendelluft (*p* = 0.042). Patients with higher defect score also had larger pendelluft amplitude [1.2 (0.5, 2.5) versus 1.5 (0.7, 3.8) versus 2.1 (0.9, 6.8) in % of global TIV for defect score 0, 1, ≥ 2, respectively; Kruskal–Wallis *p* = 0.011] (Additional file 1: Fig. S3). Subsequent multiple comparisons showed the difference was significant only between defect score 0 and ≥ 2 (*p* = 0.008).

## Outcome

**Fig. 2:**
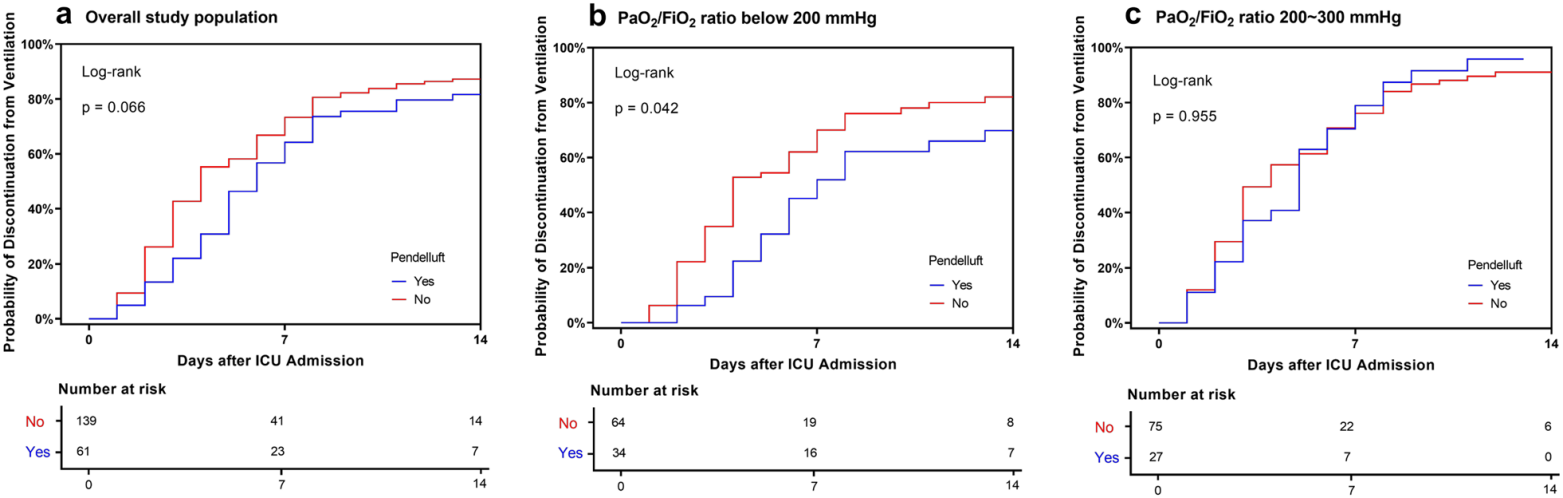
Kaplan–Meier 14-day probability of discontinuation from ventilation curve for patients with (blue) or without pendelluft (red) in **A** the overall study population, **B** in patients with PaO_2_/FiO_2_ ratio below 200 mmHg and **C** between 200 and 300 mmHg

**Fig. 3:**
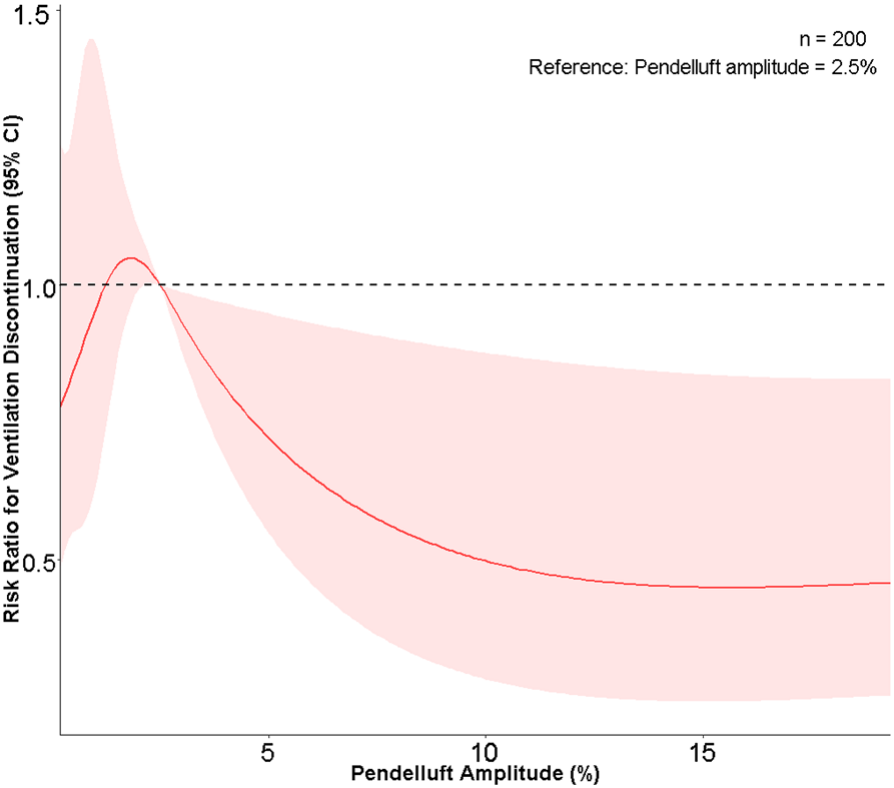
Relationship between pendelluft amplitude and risk ratio for ventilation discontinuation in the study population. 2.5% amplitude of pendelluft was set as the reference

The outcome of patients with or without pendelluft is displayed in Tables 1 and 3 and Figs. 2 and 3. ICU mortality was 12% for the whole study population and did not differ between the groups (*p* = 0.151). ICU length of stay was longer [10 (6, 14) versus 7 (4, 11) days; *p* = 0.022] and 14-day ventilator-free days was shorter [8 (1, 10) versus 10 (6, 12) days; *p* = 0.015] in patients with pendelluft. Survival analysis revealed marginally non-significant effect of pendelluft on discontinuation from ventilation within 14 days in the overall study population (log-rank *p* = 0.066). When the study population was divided into two subgroups according to PaO_2_/FiO_2_ ratio, pendelluft was associated with significantly longer 14-day ventilation duration in patients with PaO_2_/FiO_2_ ratio below 200 mmHg (log-rank *p* = 0.042) while it had no effect on 14-day ventilation duration in patients with PaO_2_/FiO_2_ ratio above 200 mmHg (log-rank *p* = 0.930). Cox regression also identified pendelluft as an independent risk factor for longer 14-day ventilation duration, after being adjusted by APACHE II score and PaO_2_/FiO_2_ ratio (Table 3). Higher pendelluft amplitude was associated with lower likelihood of discontinuing from mechanical ventilation within 14 days, taken pendelluft amplitude 2.5% as a reference (Fig. 3).

**Table 3:** Univariate and multivariate Cox regression analysis for discontinuation from ventilation at Day 14 among patients with PaO_2_/FiO_2_ ratio < 200 mmHg

Variables	Univariate Cox regression		Multivariate Cox regression	
	Hazard ratio (95% CI)	*p*	Hazard ratio (95% CI)	*p*
Pendelluft	0.592 (0.354–0.991)	0.046	0.562 (0.334–0.946)	0.030
APACHE II score	0.930 (0.891–0.970)	0.001	0.922 (0.882–0.964)	< 0.001
PaO_2_/FiO_2_ ratio	1.008 (1.002–1.015)	0.016	1.009 (1.002–1.016)	0.015
PEEP	1.020 (0.980–1.122)	0.684	Not included	

*APACHE* Acute Physiology and Chronic Health Evaluation, *PEEP* positive end-expiratory pressure, *PaO*_*2*_ arterial partial pressure of oxygen, *FiO*_*2*_ fraction of inspired oxygen

Outcome analyses were also performed in patients with and without spontaneous breathing separately (Additional file 1: Fig. S4). In either group, pendelluft was not associated with longer 14-day ventilation duration, but further investigation into patients with spontaneous breathing and simultaneously P/F ratio below 200 mmHg showed a marginal effect of pendelluft with longer 14-day ventilation duration (log rank *p* = 0.081).

## Discussion

The main findings of our study were that: (1) pendelluft was detected in 31% of 200 ARF patients ventilated in the ICU; (2) higher GI index and the existence of spontaneous breathing were the independent factors associated with pendelluft; (3) pendelluft was associated with longer 14-day ventilation duration among patients with PaO_2_/FiO_2_ ratio below 200 mmHg.

### Definition and prevalence of pendelluft

Since the first report of “occult pendelluft” phenomenon in anesthetized pigs with acute lung injury and a patient with acute respiratory distress syndrome (ARDS) in 2013 [3], EIT has been increasingly used to detect the pendelluft in critically ill patients [14–17]. At least two studies tried to quantitatively assess the gas volume subjected to pendelluft and moving within the lungs through EIT-based algorithms. Coppadoro et al. [8] defined the increased regional impedance from four quadrant ROIs during the global expiratory phase and vice versa as pendelluft. They reported a median pendelluft volume of 3.3 (2.1, 8.8) mL in 20 patients who had just failed a spontaneous breathing test. Sang et al. [9] introduced a method to detect the amplitude of pendelluft by comparing the sum of all pixel TIV with the global TIV, and expressed it as percent, where 1% of pendelluft amplitude was equal to 1 mL pendelluft volume per 100 mL tidal volume. The latter algorithm was closer to the original theory of pendelluft proposed by Otis et al. [11] and was adopted in our study. According to Otis et al., pendelluft could occur where heterogeneity of respiratory time constants (compliance * resistance) existed between adjacent alveoli. The time shift due to heterogeneity of time constants within the lungs could be assessed by the present pendelluft evaluation. On the other hand, heterogeneous time constants may also result in a so-called “regional ventilation delay”: heterogeneous regional inflation as compared to the global due to collapse of alveoli and/or airways without time shift at the end of inspiration. Such regional ventilation delay was not captured by our calculation. Although the definition of pendelluft was relatively clear, the problem was, the EIT-based algorithm was so sensitive to detect a small amount of pendelluft, possibly without pathological significance, in healthy adults without underlying lung diseases. Therefore, we investigated the pendelluft amplitude in 30 healthy volunteers and then set the 95th percentile, i.e., 2.5% as a threshold, only above which the occurrence of clinically significant pendelluft was considered. Based on that definition, we were able to report the incidence of pendelluft for the first time among ventilated ICU patients with ARF.

### Risk factors for pendelluft

Respiratory time constants inequality, also interpreted as alveolar heterogeneity, is the basis of respiratory pendelluft. In the present study, we used two easily accessible parameters to describe the lung heterogeneity. One was the ventilation defect score [12], a semi-quantitative parameter to describe the severity of uneven gas distribution to four quadrant ROIs, ranging from 0 to 6. Higher scores, reflecting higher heterogeneity in gas distribution, were associated with larger amplitudes of pendelluft. The other was the GI index [13], widely used in the evaluation of lung recruitment, PEEP titration and weaning process. The calculation of the GI index was based on the deviation of each pixel tidal impedance variation. Higher GI values denoted higher degree of lung heterogeneity. Our study recognized GI index as an independent factor associated with pendelluft.

Spontaneous breathing effort during mechanical ventilation might improve gas exchange and lung aeration, but excessive effort could also cause uneven distribution of intrathoracic pressure in already injured lung, which was proposed as another mechanism eliciting respiratory pendelluft [3]. Previous studies noticed the disappearance of pendelluft in ARDS patients when neuromuscular blockers were applied [3, 18], supporting the association between spontaneous effort and pendelluft. In our study population, pendelluft was more likely to occur in patients with spontaneous breathing, but there was no direct evaluation of breathing effort. Respiratory rate was an indirect indicator reflecting the breathing effort [19, 20]. It was higher in patients with pendelluft but without statistical significance after multivariable regression. Relatively low respiratory frequency (below 21 cycles/minute in 75% of spontaneously breathing patients) might obscure its effect. Careful monitoring of spontaneous breathing effort (e.g., P0.1, pressure muscle index, negative deflection of pressure during end-expiratory occlusion, etc.) and its association with pendelluft amplitude is needed. It should be also noted that zero spontaneous effort did not exclude the possibility of pendelluft, as was seen in around 21% of ARF patients without presence of spontaneous breathing and was proven by dynamic computed tomography in an experimental study conducted on a swine model of mild acute respiratory distress syndrome [18].

Some studies found that applying higher PEEP could alleviate pendelluft in ARDS [4, 5]. As pendelluft was mainly associated with lung heterogeneity and dynamic pleural pressure variations, higher PEEP may reduce the magnitude of pendelluft by lowering the level of spontaneous effort via neuromechanical uncoupling and by reducing atelectasis. Both mechanisms promote a more homogeneous lung expansion [21]. Opposite evidence also existed showing PEEP had no effect on pendelluft [22], but the ventilation mode, baseline P/F ratio and calculation of pendelluft in the study were all different from previous ones. Hence, we made subgroup analysis and found a week but significant negative correlation between PEEP and pendelluft amplitude in the more hypoxemic population and patients with spontaneous breathing. The different results among studies could be partly explained by the disease severity and whether spontaneous breathing was present.

### Clinical implication and prognosis of pendelluft

Pendelluft has the potential to cause lung injury as it could increase local lung stress and cause regional overdistension even under protective ventilator settings. Previous animal experiments suggested that pendelluft was associated with tidal recruitment, and that effort-dependent lung injury occurred in the same region where pendelluft appeared [4–6]. Our study revealed for the first time that pendelluft was associated with longer duration of mechanical ventilation among ICU patients with PaO_2_/FiO_2_ ratio below 200 mmHg, after APACHE II score and PaO_2_/FiO_2_ ratio was adjusted. The effect of pendelluft on ventilation duration was dependent on the severity of ARF as similar effect was not seen in mild impaired oxygenation. Results from subgroup analysis according to whether spontaneous breathing was present might suggest different clinical impact of pendelluft in active or passive condition (Additional file 1: Fig. S4). We supposed that pendelluft associated with spontaneous effort in patients with moderate-to-severe impaired oxygenation could be an injurious ventilation pattern that possibly lengthen ventilation duration, while pendelluft in passive condition might only imply lung inhomogeneity but no direct evidence to lung injury.

Results from analysis of restricted cubic spline suggested that pendelluft amplitude below the reference of 2.5% had an unclear influence on the probability of successful discontinuation from mechanical ventilation, while higher amplitude of pendelluft above the threshold was associated with prolonged ventilation duration. The optimal threshold of pendelluft amplitude or volume for predicting clinical outcome warrants further investigation.

We also explored the relationship between pendelluft amplitude and ventilatory ratio, a variable reflecting ventilation efficiency of the lung [10]. A positive correlation was hypothesized because the pendelluft gas moving within the lung was not expected to contribute to gas exchange, possibly resulting in reduction of ventilation efficiency. However, our results did not support the hypothesis. The effect of pendelluft on ventilation efficiency might have been too weak or masked by confounders.

## Study limitations

Our study has some limitations. First, the overall study population was heterogeneous, with both assisted and controlled ventilation. The subjects had only mild-to-moderate impaired oxygenation on average, which might weaken the effect of pendelluft on clinical outcome. That was also the reason why we chose 14-day rather than 28-day ventilator-free day as the primary mechanical ventilation-related outcome. Second, the out-of-phase impedance change generated by diaphragm movement [23] or pleural effusion [24] could be mistaken as pendelluft. Since an upward shift of diaphragm was common in patients with obesity or increased intra-abdominal pressure, electrode belts were placed at a higher position in these patients (3–4th intercostal space) according to their chest X-ray or lung ultrasound findings to avoid the signal interference from the moving diaphragm. Future EIT algorithm needs be updated to distinguish lung regions with present diaphragm movement or pleural effusion. Third, the respiratory management was probably influenced by EIT results in some cases, introducing a potential bias when assessing the impact of pendelluft on patient outcome.

## Conclusions

In conclusion, pendelluft was identified in 31% of a single-center ARF patients ventilated in the ICU. Pendelluft occurred more often in cases with spontaneous breathing and higher lung heterogeneity. It was associated with longer ventilation duration in patients with PaO_2_/FiO_2_ ratio below 200 mmHg. Careful monitoring and therapies aimed at alleviating pendelluft should be tested in patients with severe hypoxia in the future.

## Supplementary Information

**Additional file 1:** **Figure S1.** Schematic diagram of EIT-measured pendelluft amplitude. Pixel 1 and 2 are impedance-time curves from two representative pixels with large ventilation shift. The EIT-based pendelluft amplitude is calculated as the impedance difference between the sum of all pixel TIV and the global TIV. TIV, tidal impedance variation. A.U., arbitrary unit. **Figure S2.** Schematic diagram of ventilation defect score. **Figure S3.** Relationship between ventilation defect score and pendelluft amplitude. **Figure S4.** Survival analyses performed in patients with and without spontaneous breathing separately. Kaplan–Meier 14-day probability of discontinuation from ventilation curve for patients with (blue) or without pendelluft (red) in the patients **A** with spontaneous breathing, **B** absence of spontaneous breathing, **C** spontaneous breathing and P/F ratio below 200 mmHg and **D** absence of spontaneous breathing and P/F ratio below 200 mmHg

## Data Availability

The datasets used or analyzed in the study are available from the corresponding author on reasonable request.

## Abbreviations

APACHE: Acute Physiology and Chronic Health Evaluation
ARDS: Acute respiratory distress syndrome
ARF: Acute respiratory failure
CI: Confidence interval
EIT: Electrical impedance tomography
FiO_2_: Fraction of inspiratory oxygen
GI: Global inhomogeneity
ICU: Intensive care unit
LL: Lower left
LR: Lower right
MV: Mechanical ventilation
PBW: Predicted body weight
PaO_2_: Partial pressure of arterial oxygen
PEEP: Positive end-expiratory pressure
ROI: Region of interest
TIV: Tidal impedance variation
UL: Upper left
UR: Upper right
VFD: Ventilator-free day
