# Supplementary material for: Prevalence and prognosis of respiratory pendelluft phenomenon in mechanically ventilated ICU patients with acute respiratory failure: a retrospective cohort study
Source: Ann Intensive Care. 2022 Mar 5;12:22. doi: 10.1186/s13613-022-00995-w (PMC8897528; doi:10.1186/s13613-022-00995-w)
Supplement: Supplementary file 1 — Additional file 1: Figure S1. Schematic diagram of EIT-measured pendelluft amplitude. Pixel 1 and 2 are impedance-time curves from two representative pixels with large ventilation shift. The EIT-based pendelluft amplitude is calculated as the impedance difference between the sum of all pixel TIV and the global TIV. TIV, tidal impedance variation. A.U., arbitrary unit. Figure S2. Schematic diagram of ventilation defect score. Figure S3. Relationship between ventilation defect score and pendelluft amplitude. Figure S4. Survival analyses performed in patients with and without spontaneous breathing separately. Kaplan–Meier 14-day probability of discontinuation from ventilation curve for patients with (blue) or without pendelluft (red) in the patients A with spontaneous breathing, B absence of spontaneous breathing, C spontaneous breathing and P/F ratio below 200 mmHg and D absence of spontaneous breathing and P/F ratio below 200 mmHg [file 13613_2022_995_MOESM1_ESM.docx]

**Table S1.** Ventilation distribution and pendelluft amplitude from 30 healthy volunteers without underlying lung diseases.

| **Number** | **Gender** | **Age (years)** | **Height (cm)** | **Weight (kg)** | **ROI-UR%** | **ROI-UL%** | **ROI-LR%** | **ROI-LL%** | **Defect Score** | **Pendelluft Amplitude (%)** | **GI index** |
| --- | --- | --- | --- | --- | --- | --- | --- | --- | --- | --- | --- |
| 1 | male | 30 | 165 | 70 | 28 | 15 | 29 | 28 | 0 | 0.9 | 0.35 |
| 2 | male | 35 | 170 | 65 | 25 | 35 | 20 | 21 | 0 | 0.3 | 0.33 |
| 3 | female | 21 | 172 | 70 | 22 | 35 | 24 | 19 | 0 | 0.6 | 0.34 |
| 4 | male | 26 | 180 | 72 | 30 | 19 | 35 | 16 | 0 | 1.4 | 0.31 |
| 5 | female | 28 | 183 | 80 | 30 | 26 | 21 | 22 | 0 | 0.3 | 0.29 |
| 6 | female | 42 | 159 | 60 | 27 | 15 | 35 | 24 | 0 | 0.3 | 0.36 |
| 7 | male | 27 | 188 | 74 | 27 | 20 | 33 | 20 | 0 | 0.5 | 0.33 |
| 8 | female | 25 | 168 | 55 | 32 | 29 | 17 | 22 | 0 | 0.5 | 0.33 |
| 9 | male | 34 | 186 | 75 | 34 | 31 | 18 | 17 | 0 | 0.6 | 0.31 |
| 10 | male | 25 | 179 | 55 | 25 | 27 | 26 | 21 | 0 | 2.1 | 0.4 |
| 11 | female | 44 | 158 | 58 | 23 | 23 | 30 | 23 | 0 | 1.2 | 0.34 |
| 12 | male | 35 | 168 | 80 | 31 | 34 | 20 | 16 | 0 | 1.2 | 0.33 |
| 13 | male | 32 | 181 | 68 | 24 | 19 | 29 | 29 | 0 | 1.9 | 0.38 |
| 14 | female | 39 | 179 | 78 | 24 | 26 | 25 | 25 | 0 | 5.4 | 0.38 |
| 15 | male | 37 | 178 | 80 | 26 | 24 | 30 | 19 | 0 | 1.3 | 0.33 |
| 16 | female | 31 | 175 | 95 | 21 | 31 | 23 | 25 | 0 | 0.6 | 0.33 |
| 17 | male | 33 | 179 | 98 | 34 | 42 | 12 | 12 | 2 | 2.1 | 0.35 |
| 18 | female | 29 | 160 | 59 | 21 | 24 | 31 | 25 | 0 | 0.5 | 0.33 |
| 19 | male | 34 | 157 | 52 | 24 | 23 | 27 | 25 | 0 | 2.8 | 0.39 |
| 20 | female | 22 | 158 | 53 | 25 | 19 | 29 | 27 | 0 | 0.4 | 0.34 |
| 21 | female | 28 | 164 | 53 | 21 | 28 | 21 | 30 | 0 | 0.5 | 0.32 |
| 22 | female | 31 | 160 | 56 | 22 | 19 | 30 | 28 | 0 | 0.5 | 0.36 |
| 23 | male | 50 | 160 | 60 | 29 | 29 | 22 | 20 | 0 | 0.7 | 0.36 |
| 24 | female | 35 | 165 | 56 | 20 | 21 | 33 | 26 | 0 | 0.6 | 0.32 |
| 25 | male | 32 | 168 | 51 | 19 | 24 | 31 | 26 | 0 | 0.5 | 0.32 |
| 26 | male | 26 | 163 | 54 | 18 | 31 | 28 | 22 | 0 | 0 | 0.35 |
| 27 | female | 38 | 150 | 50 | 27 | 23 | 23 | 26 | 0 | 0.6 | 0.37 |
| 28 | female | 28 | 172 | 60 | 22 | 25 | 25 | 29 | 0 | 0.4 | 0.33 |
| 29 | male | 29 | 172 | 52 | 25 | 25 | 30 | 21 | 0 | 1.1 | 0.37 |
| 30 | female | 41 | 166 | 68 | 28 | 19 | 33 | 21 | 0 | 2 | 0.36 |

ROI = region of interest; UL: Upper left; UR: Upper right; LL = Lower left; LR = Lower right; GI = global inhomogeneity.

**Figure S1.** Schematic diagram of EIT-measured pendelluft amplitude.


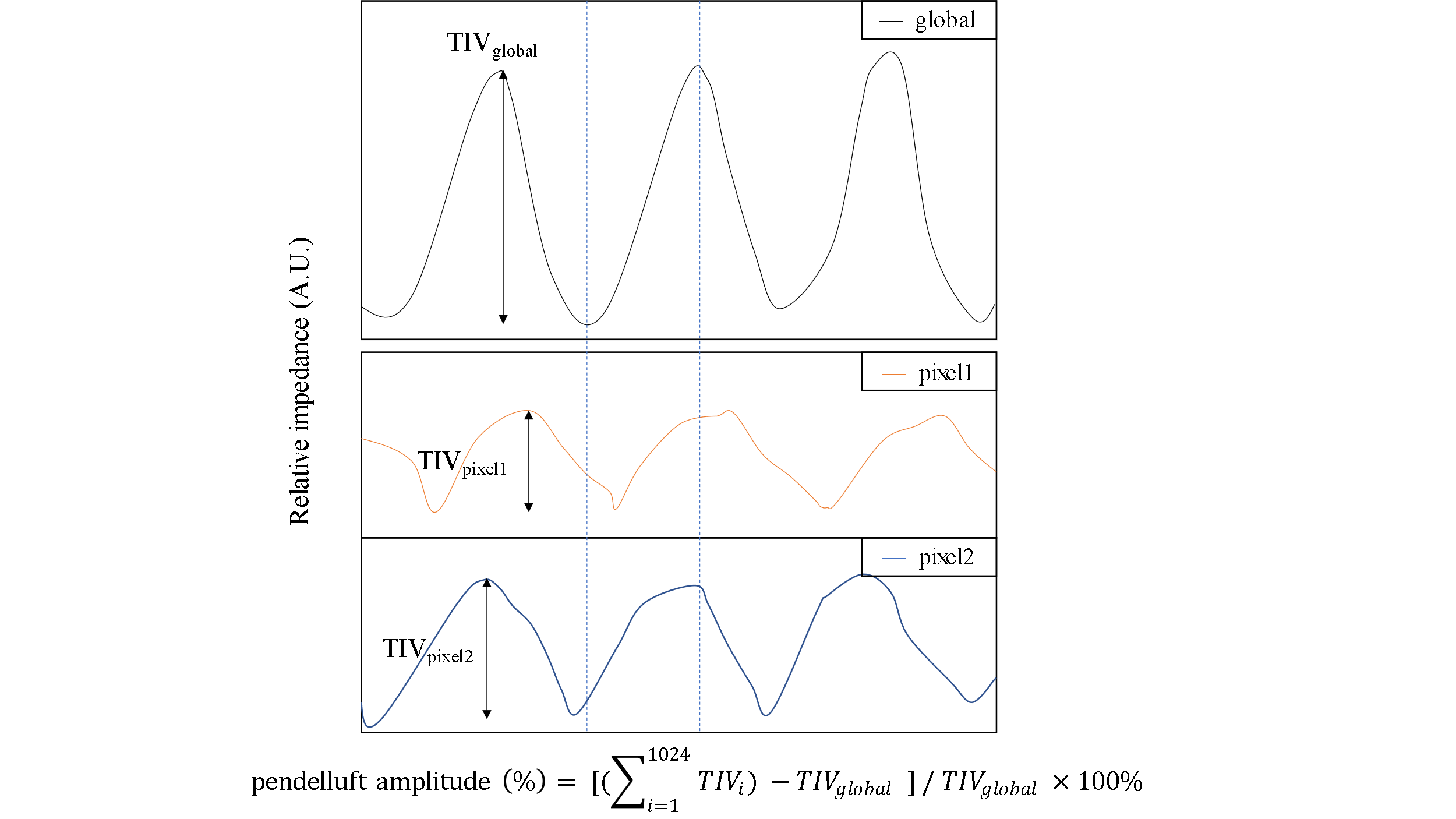


Pixel 1 and 2 are impedance-time curves from two representative pixels with large ventilation shift. The EIT-based pendelluft amplitude is calculated as the impedance difference between the sum of all pixel TIV and the global TIV. TIV, tidal impedance variation. A.U., arbitrary unit.

**Figure S2.** Schematic diagram of ventilation defect score.


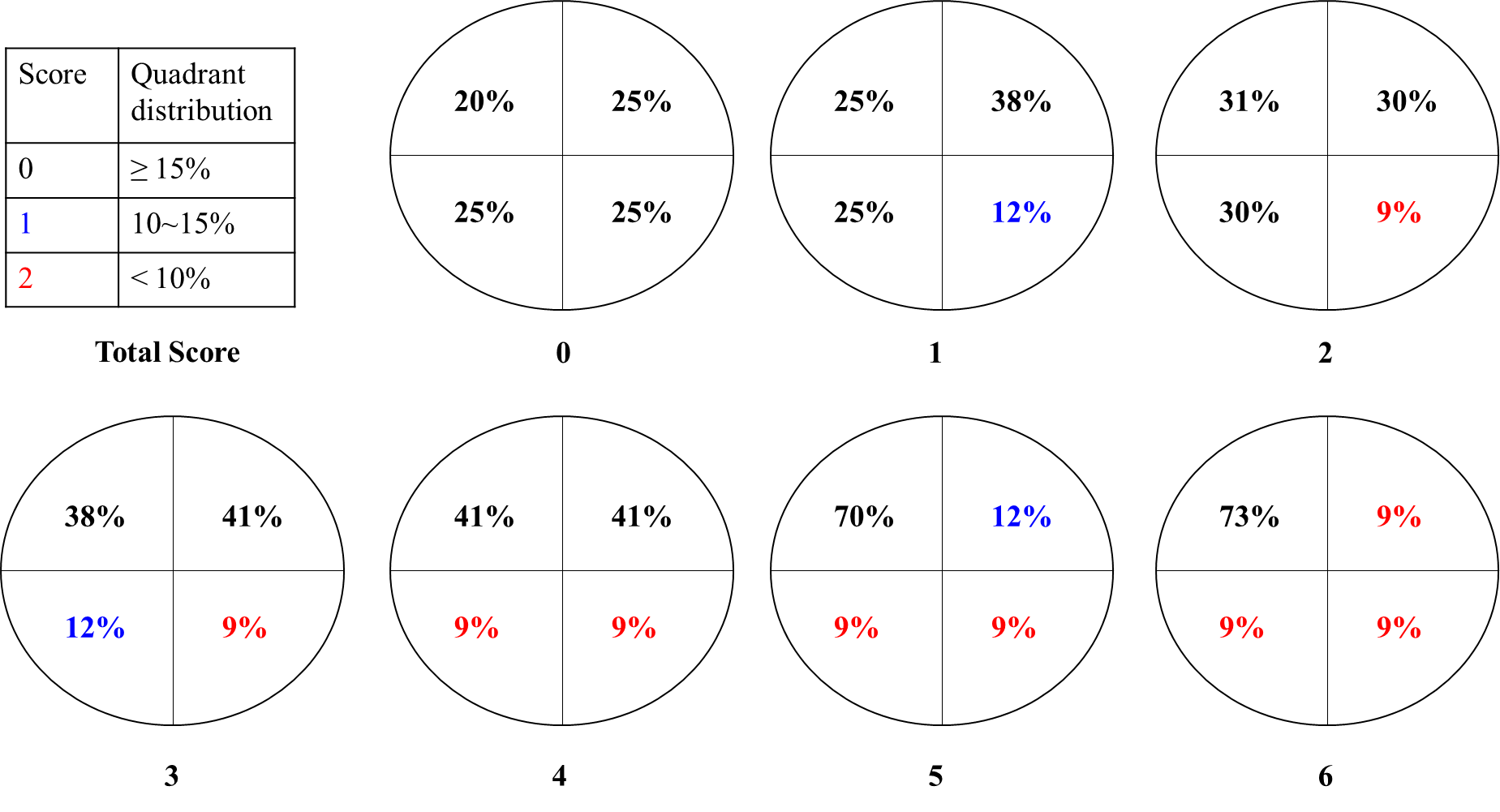


**Figure S3.** Relationship between ventilation defect score and pendelluft amplitude.

**
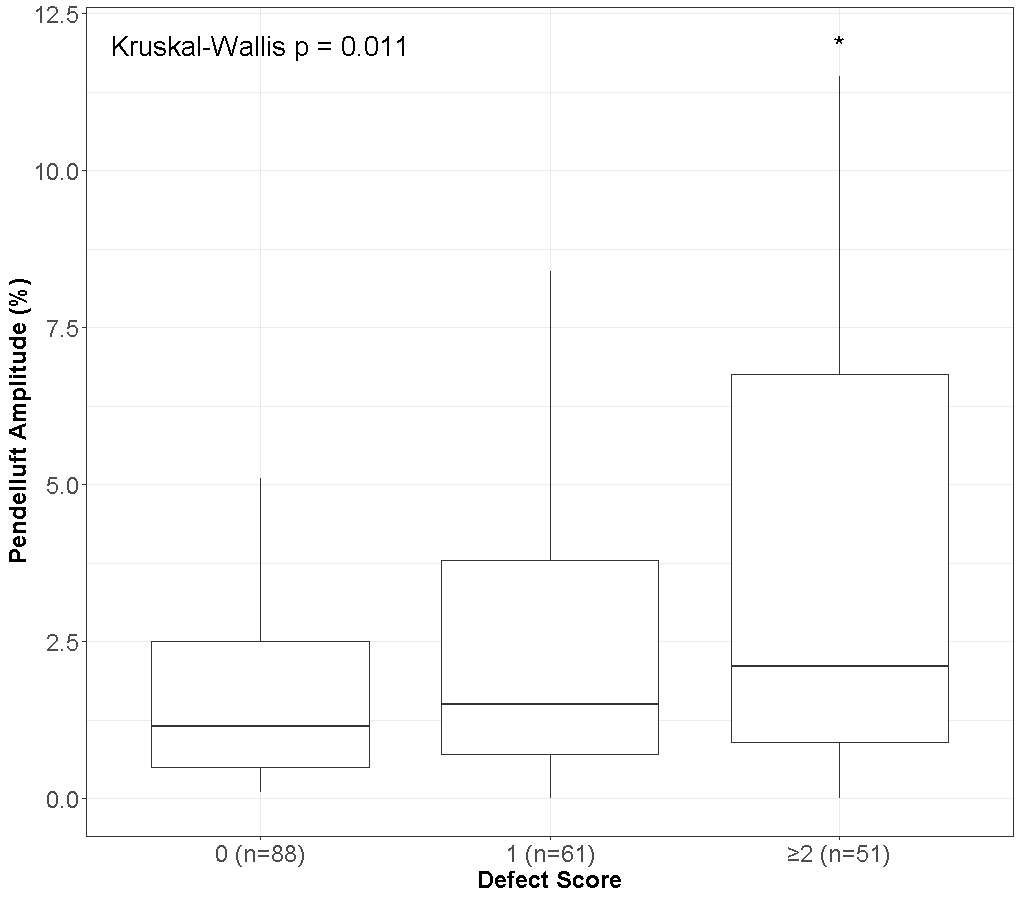
**

* p<0.05 compared with “defect score = 0”.

**Figure S4.** Survival analyses performed in patients with and without spontaneous breathing separately.


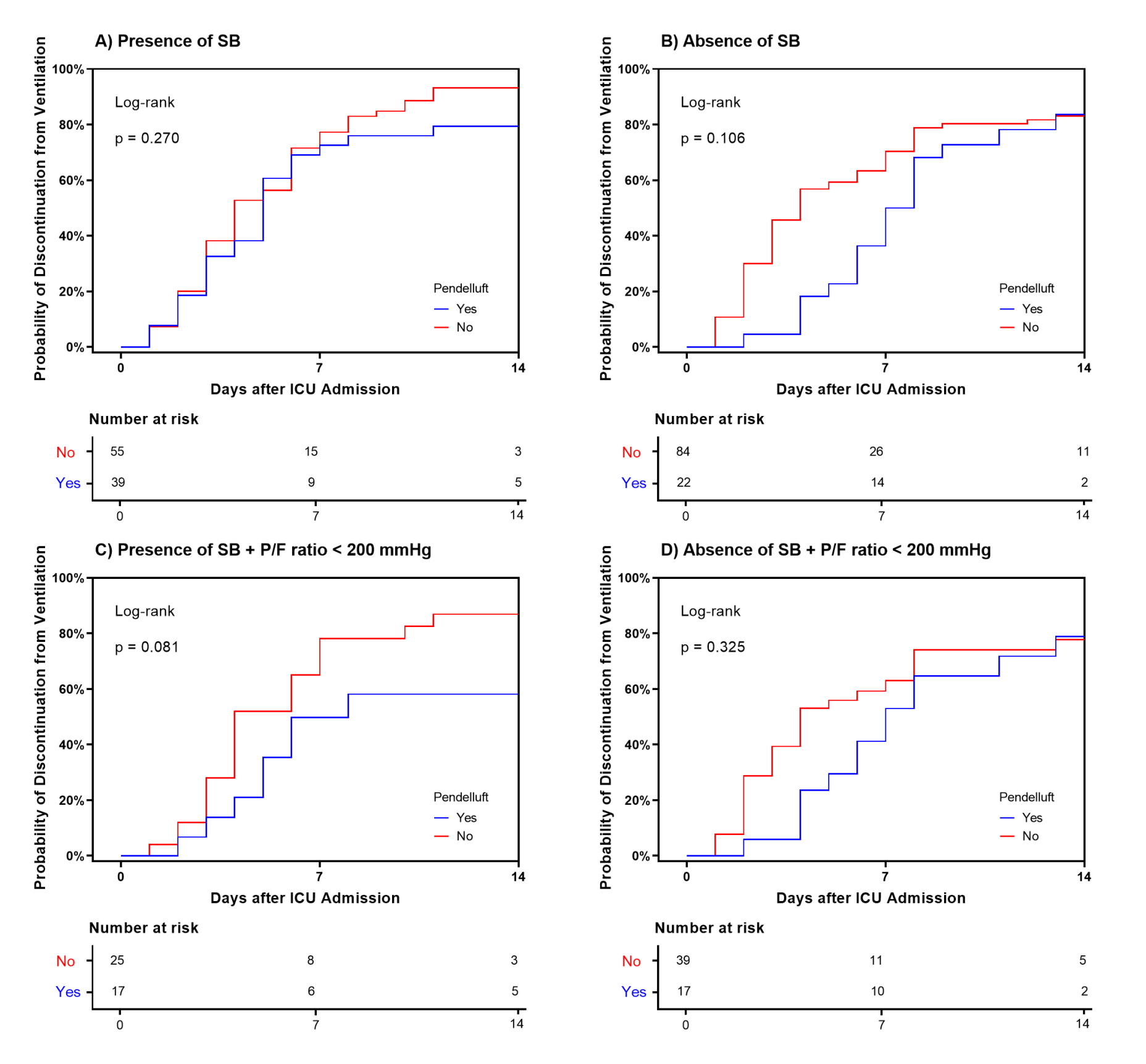


Kaplan–Meier 14-day probability of discontinuation from ventilation curve for patients with (blue) or without pendelluft (red) in the patients **A)** with spontaneous breathing, **B)** absence of spontaneous breathing, **C)** spontaneous breathing and P/F ratio below 200 mmHg and **D)** absence of spontaneous breathing and P/F ratio below 200 mmHg**.**
